# The Fatty Acid Profile of Meat from New Zealand White Rabbits Raised under Intensive and Extensive Production Systems

**DOI:** 10.3390/ani11113126

**Published:** 2021-10-31

**Authors:** Tomasz Daszkiewicz, Andrzej Gugołek, Dorota Kubiak, Krzysztof Kerbaum, Ewa Burczyk

**Affiliations:** 1 Department of Commodity Science and Processing of Animal Raw Materials, University of Warmia and Mazury in Olsztyn, Oczapowskiego 5, 10-719 Olsztyn, Poland; dorota.kubiak@uwm.edu.pl (D.K.); kkerbaum@wp.pl (K.K.); ewabur@uwm.edu.pl (E.B.); 2 Department of Fur-Bearing Animal Breeding and Game Management, University of Warmia and Mazury in Olsztyn, Oczapowskiego 5, 10-719 Olsztyn, Poland; gugolek@uwm.edu.pl

**Keywords:** rabbits, production system, meat, fatty acid profile

## Abstract

**Simple Summary:**

The intensification of livestock production in response to the growing demand for food has disrupted the balance between the quantity and quality of animal-based products, contributing to changes in consumer preferences. Modern consumers show an increasing interest in extensive farming and niche products that are regarded as more unique, tastier, and healthier due to the absence of harmful compounds and a higher content of valuable components (vitamins, micronutrients, macronutrients, unsaturated fatty acids). However, consumers often formulate their views and opinions based on widely-held beliefs that are not always supported by research findings. Therefore, science-based evidence is needed to draw reliable conclusions about meat products. The fatty acid composition of meat from rabbits raised under intensive and extensive production systems was compared in this study. It was found that intensively farmed rabbits can produce meat of superior quality.

**Abstract:**

The aim of this study was to compare the fatty acid (FA) profile of meat from New Zealand White rabbits raised from 30 to 90 days of age under intensive (IPS) and extensive (EPS) production systems. In group IPS, the rabbits were housed in wire mesh cages with a slatted floor (16.7 animals/m^2^) and were fed a commercial pelleted diet. In group EPS, the rabbits were housed in free-standing cages on straw litter (2.5 animals/m^2^) and were fed a conventional farm-made diet (green fodder, barley grain, stale bread, hay). The FA profile of intramuscular fat (IMF) was analyzed in samples of *Longissimus thoracis et lumborum* (LTL) muscle. The analyzed production systems had no significant effect on the content of most FAs in IMF. However, the differences between group means contributed to more desirable values of the quality indicators of IMF (in particular in the LTL muscle) in group IPS. The study demonstrated that the claim that meat produced under less intensive farming systems is of superior quality could be an oversimplified generalization that should be validated in research.

## 1. Introduction

In recent decades, the high demand for food on global markets has contributed to the intensification and standardization of food production. In the coming years, this trend is likely to be maintained due to rapid population growth [1]. However, consumers in highly developed countries show a growing interest in high-quality foods [2,3]. As a result, food suppliers are beginning to diversify products to cater to the needs and expectations of various consumer groups. These marketing strategies involve the introduction of niche foods, organic products, and foods manufactured in less intensive production systems. The demand for foods from extensive production systems continues to increase because contemporary consumers are mistrustful of foods produced in traditional agricultural systems, where the main emphasis is placed on quantity rather than quality. This trend can be attributed to growing levels of awareness about the impact of intensive crop and livestock production on the quality and health benefits of raw materials and end products [4]. Many consumers believe that foods produced under intensive farming systems contain more harmful substances, have inferior sensory attributes, are deficient in valuable nutrients (vitamins, minerals, unsaturated fatty acids—UFAs), contribute to environmental degradation, and compromise animal welfare [5]. These beliefs increase the demand for “sustainable” foods originating from less intensive production systems.

Rabbits are generally regarded as an animal species that are typically raised in small farms and traditional production systems [6]. Meanwhile, rabbits are raised for meat in many countries and regions of the world. In 2016, global rabbit meat production reached 1,428,085 tons [7], where Asia, Europe, Africa, and America were responsible for around 73%, 20%, 6%, and 1% of the global production, respectively [8]. In the EU, rabbits are the second most farmed species in terms of numbers [9], where 34% of 180 million animals raised for meat are kept in family farms [10]. Most of these farms are small and medium-sized enterprises where rabbits are produced less intensively than in large commercial farms. Extensive farming differs from intensive production systems, mainly in housing conditions and animal diets. In extensive farms, rabbits are kept on litter in cages or pens, movable outdoor pens with access to indoor and outdoor areas, or even outdoors. Rabbits are fed farm-made diets, and the use of chemotherapeutics is limited. In many respects, extensive farming is similar or equivalent to organic farming [11].

Modern rabbit breeds differ in their suitability for extensive and intensive production [12]. The following rabbit breeds are most widely farmed for meat: native (local) breeds, colored (extensive) breeds, intensive breeds (such as New Zealand and Californian), synthetic breeds, and hybrids [13]. Native and colored breeds are best suited for extensive production, but intensive breeds can also be used in these production systems. However, the conditions under which intensive breeds are farmed under extensive production systems have to be optimized to guarantee production success.

Extensive (and organic) farming methods are highly varied, and they are difficult to compare with intensive production systems in terms of output [14]. The limited number of research studies involving such comparisons confirms the above observation. However, objective research findings are needed to shape consumer attitudes and undermine subjective and stereotypical beliefs about the superior quality of foods produced in less intensive systems. Due to considerable variations in production factors in extensive farming systems, the quality of the resulting products is not always superior to that noted in properly managed intensive systems. The above hypothesis could also apply to the fatty acid (FA) profile of rabbit meat which plays a significant role in human nutrition [15].

The aim of this study was to compare the FA profile of meat from New Zealand White rabbits raised under intensive and extensive production systems.

## 2. Materials and Methods

The experiment was approved by the local Ethics Committee for Animal Experimentation (Olsztyn, Poland, decision No. 24/2014) as part of long-term comprehensive studies investigating the factors that affect the quality of rabbit meat. The experiment was conducted in spring and summer, in two rabbit farms (A and B) located in north-eastern Poland, on 40 New Zealand White rabbits born in farm A. The animals were weaned at 30 days of age, and they were divided into a group representing an intensive production system (IPS) and a group representing an extensive production system (EPS). Throughout the experiment, group ISP rabbits were kept on farm A, in wire mesh cages with a slatted floor (0.4 m × 0.6 m × 0.32 m), with 4 animals per cage (16.7 animals/m^2^), indoor (natural lighting, temperature of 15–20 °C). They were fed *ad libitum* a commercial pelleted diet (wheat bran—36%, dehydrated alfalfa meal—25%, dried beet pulp—10%, sunflower husk—6%, rapeseed meal—5%, soybean meal—4%, barley—4%, palm cake—3%, rye bran—3%, vitamin-mineral premix—1%, lime—1%, dicalcium phosphate—0.5%, salt—0.5%), supplemented with the coccidiostat robenidine (robenidine hydrochloride—65 mg/kg). The chemical composition and FA profile of the experimental diet are presented in Table 1.

Group EPS rabbits were transported to a small-scale farm (B), located at a distance of 25 km from farm A, where they were housed in free-standing cages (1 m × 2 m × 0.6 m) on wheat straw litter, with 5 animals per cage (2.5 animals/m^2^). They were fed *ad libitum* a conventional, farm-made diet composed of green fodder (70%), barley grain (10%), stale bread (10%), and hay (10%). Feed was supplied twice daily, and the daily ration was increased as the animals grew older, taking into account the number of leftovers. Group EPS rabbits did not receive any coccidiostat. The chemical composition and FA profile of the experimental diet are presented in Table 1. Both diets were formulated to meet the nutrient requirements of rabbits [16].

The rabbits (10 males from groups IPS and EPS) were slaughtered at 91 days of age, after 24 h of fasting. Chilled carcasses (0–2 °C, 24 h) were divided into the following parts: head (cut through the atlanto-occipital joint), fore part (cut between the last thoracic vertebra and the first lumbar vertebra), intermediate part (cut behind the last lumbar vertebra) and hind part (carcass section that remains after the intermediate part had been cut off the fore part). The *Longissimus thoracis et lumborum* (LTL) muscle was removed from the intermediate part, and leg muscles (LM) were removed from the hind part of each carcass. The muscles were transported to the laboratory, where they were passed three times through a 3 mm plate in a meat grinder and thoroughly mixed to obtain samples for analyses.

The FA profile was determined in intramuscular fat (IMF) extracted from LTL muscle and LM samples by the Soxhlet method [17]. Fatty acid methyl esters were extracted by the modified method of Peisker [18]. Fatty acids were separated by gas chromatography on the VARIAN CP-3800 gas chromatograph (Varian Inc., Palo Alto, CA, USA), coupled to a flame ionization detector (FID) and a capillary column (length—50 m, inner diameter—0.25 mm, liquid phase—CP-Sil 88, film thickness—0.25 μm). The carrier gas was helium (flow rate—1.2 mL/min). Fatty acids were identified by comparing the retention times of methyl esters in the analyzed samples and the standard mixture of fatty acid methyl esters (Sigma, St. Louis, MO, USA). The results were presented as percentages of individual FAs in total FAs in IMF.

### Statistical Analysis

The results were analyzed statistically with the use of STATISTICA ver. 13.3 software (TIBCO Software Inc., Palo Alto, CA, USA). The effect of the experimental factor (IPS/EPS) on the FA profile of rabbit meat (in the LTL muscle and LM) was determined by one-way analysis of variance (ANOVA). The significance of differences between group means was estimated by Bonferroni’s test at *p* ≤ 0.05.

## 3. Results

The SFA profile of IMF is presented in Table 2. The average concentrations of individual SFAs in the LTL muscle and LM of rabbits were similar in both IPS and EPS. Production system affected only the content of pentadecanoic acid (C15:0), which was higher (*p* = 0.018) in the LTL muscle of rabbits in group EPS.

The meat of extensively farmed rabbits had higher (but not statistically significant—*p* > 0.05) content of lauric acid (C12:0) (LTL—*p* = 0.468, LM—*p* = 0.192), myristic acid (C14:0) (LTL—*p* = 0.268, LM—*p* = 0.149), and palmitic acid (C16:0) (LTL—*p* = 0.179, LM—*p* = 0.448), as well as stearic acid (C18:0) in the LTL muscle (*p* = 0.131). As a result, the muscles of extensively farmed rabbits had a higher total content of SFAs, but a significant (*p* = 0.039) difference between group means was noted only in the LTL muscle.

The UFA profile of IMF is presented in Table 3. In the group of MUFAs, production system affected only the content of palmitoleic acid (C16:1), which was higher (*p* = 0.003) in the LTL muscle of rabbits in group ESP. The concentrations of individual MUFAs and, consequently, their total content were comparable in the LTL muscle (*p* = 0.798) and LM (*p* = 0.467) of rabbits raised under both production systems.

An analysis of the content of PUFAs in rabbit meat revealed that it was higher in group IPS (Table 3). However, a significant difference between group means (*p* < 0.001) was noted only in the content of arachidonic acid (C20:4) in the LTL muscle. The differences in the concentrations of individual MUFAs in the muscles of rabbits raised under two production systems resulted in their higher total content (including the higher total content of UFAs) in the IMF of intensively farmed rabbits. The differences between group means, determined in the LTL muscle, were significant at *p* = 0.019 and *p* = 0.039, respectively.

The indicators characterizing the nutritional value of IMF were calculated based on its FA profile (Table 4). The values of these parameters were more favorable in group IPS, particularly in the LTL muscle where IMF had higher values of the UFA/SFA (*p* = 0.045) and PUFA/SFA (*p* = 0.026) ratios, a more desirable ratio of hypocholesterolemic to hypercholesterolemic FAs (DFA/OFA) (*p* = 0.048), and a higher proportion of essential FAs (EFAs) (*p* = 0.037). The MUFA/SFA ratio (LTL—*p* = 0.202, LM—*p* = 0.287) and the nutritional value of IMF (LTL—*p* = 0.361, LM—*p* = 0.209) were similar in rabbits raised under both production systems.

## 4. Discussion

Different rabbit production systems are difficult to compare due to the presence of numerous factors and variables specific to each system (genotype, gender, weaning age, slaughter age, stocking density, group size, floor type, straw litter, etc.) and their interactions [19]. Such differences were also observed in this study, where rabbits were raised under intensive and extensive conditions. Therefore, the present analysis could not provide unambiguous results, in contrast to experiments where the number of experimental factors was smaller, and their effects could be analyzed separately [3,14]. Nevertheless, since farming practice involves both intensive and extensive production, attempts are being made to compare both systems, and the obtained results can pave the way for further research investigating the effects of individual factors on the analyzed traits and parameters. However, studies into the effect of intensive and extensive production systems on the lipid composition of rabbit meat are scant. For instance, Pla [14] compared the effects of conventional and organic systems, including organic feeding, on the quality of rabbit meat. In the conventional group, rabbits were housed in collective flat-deck cages (18.8 animals/m^2^) and were fed *ad libitum* a commercial pelleted diet. In the organic group, rabbits were housed in collective pens on straw litter (5 animals/m^2^) and were fed *ad libitum* a mixture of organic products (70% of alfalfa hay and 5% of a commercial mineral/vitamin premix). The hind leg meat of organically produced rabbits had a lower (*p* < 0.05) content of SFAs and MUFAs (*p* < 0.001), a higher (*p* < 0.001) content of PUFAs, and a more favorable (*p* < 0.001) PUFA/SFA ratio, relative to the meat of conventionally produced rabbits. These findings differ from the results of this study in terms of SFA and MUFA concentrations in the meat of rabbits in groups ISP and ESP. According to the cited authors, the differences in the proportions of the analyzed FA groups in the meat of conventionally and organically raised rabbits could result from different feeding diets (differences in the SFA and PUFA content of diets) and/or different levels of locomotor activity.

Paci et al. [20] evaluated the effect of organic and conventional rearing systems on meat quality in a local population of grey-colored rabbits characterized by a slow growth rate. The rabbits were housed in outdoor or indoor colony cages according to the organic or conventional breeding system. The indoor colony cages (15–16 rabbits/m^2^) were located in the experimental rabbitry. The outdoor colony cages (5 rabbits/m^2^) were placed in an outdoor pen. The rabbits were fed *ad libitum* an organic (pelleted feed + alfalfa hay) or a conventional (pelleted feed) diet. The study revealed that meat (*Longissimus lumborum*) from rabbits housed indoors and fed conventional pellets was richer in PUFAs (*p* < 0.05) and poorer (*p* > 0.05) in SFAs and MUFAs, as compared with meat from rabbits housed indoors and outdoors and fed an organic diet. These findings are consistent with the results of this study in terms of the SFA and PUFA content of meat from intensively and extensively farmed rabbits.

The effect of rabbit diets on the FA profile of IMF, described by Pla [14] and Paci et al. [20], was also observed by other authors [21,22]. In the present study, different diets also affected the FA profile of meat from rabbits raised under intensive and extensive conditions. The experimental diets were characterized by different proportions of FA groups (Table 1), which were more desirable (higher concentrations of UFAs and PUFAs) in the diet fed to group IPS. In monogastric animals, fatty acid profiles of diet and meat are strongly correlated [23]. Fatty acids released during digestion are deposited in adipose tissue in unchanged form. Moreover, the diet richer in PUFAs favored the endogenous synthesis of long-chain n-3 and n-6 FAs from linolenic and linoleic acid, respectively. Therefore, the FA profile of adipose tissue is largely determined by the percentages of individual FAs in the ration, although it may also be affected by the anatomical location of fat [24].

When analyzing the influence of rabbit diets on the FA composition of meat (which was also noted in this study), attention should be paid to the fact that rabbits often eat their bedding material. According to Dal Bosco et al. [25], the consumption of straw (which contains low levels of nutrients) can contribute to lower feed intake in rabbits kept on the straw bed, compared with those housed on the wire net floor, thus affecting meat quality. Dal Bosco et al. [19] also demonstrated that the meat (*M. Longissimus thoracis et lumborum* and *M. Biceps femoris*) of rabbits housed on deep-litter straw had lower PUFA content than the meat of rabbits housed on wire- or plastic-mesh floors. Moreover, the use of litter in rabbit farming may be associated with certain health problems (mostly coccidiosis), which affect productive performance. Therefore, Lambertini et al. [26] concluded that growth performance, slaughter results, and carcass quality are on the whole better in animals raised traditionally in wire mesh cages.

According to Pla [14], the FA profile of rabbit meat can be affected by the locomotor activity of animals, which is related to the stocking density in cages and pens. Triacylglycerols are an energy reservoir in adipose tissue, and during physical activity, they are hydrolyzed to free FAs which are a fuel for working muscles. Mika et al. [27] reported that regular human physical activity contributed to reducing adipose tissue mass and improved metabolism. Prolonged exercise decreases the activity of lipoprotein lipase, thus reducing FA uptake. This results in the improvement of mitochondrial function and the upregulation of enzymes involved in PUFA metabolism. The exercise-induced changes in adipocyte metabolism are associated with modifications in FA composition. These modifications are adipose tissue depot-specific and follow different patterns in visceral and subcutaneous adipose tissue. The data on the influence of exercise on the FA profile of skeletal muscles in rabbits and other livestock species are limited. Szabo et al. [28] found that the content of certain FAs may be affected by the implementation of an exercise regimen. In the cited study, rabbits were exposed to treadmill running twice daily. After a four-week training, the proportion of oleic acid (C18:1 n-9) increased in both *M. longissimus dorsi* (MLD) and *M. vastus lateralis* (MVL) muscles, relative to the control group. However, the levels of stearic acid (C18:0) and arachidonic acid (C20:4 n-6) decreased significantly in the MVL muscle after the exercise. Changes in the FA profile resulting from the physically loaded condition showed the same tendency in both muscles, but it was more pronounced in the MVL muscle, probably because this muscle was exposed to more intensive exercise.

The potential effect of the locomotor activity of rabbits on the FA composition of their meat can be indirectly inferred from studies investigating production systems with different stocking densities. It appears that the number of animals per m^2^ may affect locomotion, but research results are inconclusive. D’Agata et al. [29] analyzed the effects of two housing systems (outdoor—colony cages at a density of 5 rabbits/m^2^ vs. indoor—colony cages at a density of 15 rabbits/m^2^) on meat quality (hind leg) in local grey-colored rabbits (agouti and wild type) and found that meat from outdoor rabbits contained fewer SFAs (*p* < 0.05) and more MUFAs (*p* < 0.01). In the group of PUFAs, differences between the two systems were noted only in the content of C20:4 n-6 and C22:5 n-3, which was higher (*p* < 0.05) in the meat of indoor rabbits. The differences could be due to the lower (by 0.28 percentage points) amount of IMF in their meat and, consequently, a higher percentage of phospholipids which, as noted by Enser [30], are richer in both n-6 and n-3 PUFAs.

Different results were reported by Cavani et al. [31], who analyzed the effect of an open-air housing system, where rabbits were reared in movable colony cages (0.17 m^2^ per rabbit) on a polyphyta natural pasture, and a conventional indoor rearing system, where rabbits were kept in conventional bi-cellular cages (0.07 m^2^ per rabbit) on the FA composition of meat. The above authors demonstrated that meat (hind leg) from rabbits of the “Leprino of Viterbo” synthetic breed reared outdoors had higher (*p* < 0.05) SFA content, lower (*p* < 0.01) MUFA content, and higher (*p* < 0.05) PUFA (including n-6 PUFA) content. The higher long-chain PUFA content of rabbit meat could be a consequence of the lower muscle fat content in meat from open-air reared rabbits compared with the indoor group. In view of the contradictory results reported by D’Agata et al. [29] and Cavani et al. [31], it is difficult to determine whether and how the FA profile of rabbit meat was affected by different stocking densities in IPS and EPS in the current study.

It appears that factors other than nutrition and locomotion (associated with different production systems) exert only limited effects on the FA profile of rabbit meat. This hypothesis is confirmed by the results of studies investigating the influence of floor type (wire mesh and plastic net) [19,32], housing system (cage and pen), and access to gnawing sticks (yes and no) [32] under identical stocking density and feeding conditions. However, the above factors could exert their effects by interacting with other variables.

## 5. Conclusions

The analyzed production systems had no significant effect on the content of most FAs in the IMF of New Zealand White rabbits. However, the differences between group means revealed certain tendencies, which were reflected in the indicators characterizing the FA profile and nutritional value of meat. Their values were more desirable in IMF extracted from the LTL muscle of intensively farmed rabbits. A tendency towards higher values of these indicators was also observed in IMF extracted from the LM in group IPS, but no significant differences were found relative to group EPS. The results of this study indicate that the claim that meat produced under less intensive farming systems is of superior quality could be an oversimplified generalization. Therefore, it should be validated in research accounting for highly differentiated conditions of extensive production, with particular emphasis on rabbit diets.

## Figures and Tables

**Table 1:** Chemical composition (% of dry matter) and fatty acid profile (% of fatty acid groups in total fatty acids) of experimental diets fed to rabbits.

Item	Production System				
	Intensive (IPS)	Extensive (EPS)			
	Commercial Pelleted Diet	Green Fodder	Barley Grain	Stale Bread	Hay
Dry matter	87.56	18.20	89.04	73.04	92.66
Ash	5.81	9.43	2.71	2.50	5.54
Total protein	16.65	16.46	12.40	12.76	11.91
Crude fat	3.13	2.55	1.95	0.40	1.46
NDF	24.84	42.34	30.64	0.53	56.45
ADF	15.45	26.98	7.35	0.66	36.23
ADL	4.22	2.19	1.09	0.81	4.82
SFAs	20.35	27.48	24,31	20.83	37.00
UFAs	79.65	72.52	75.69	79.17	63.00
MUFAs	19.36	13.00	17.55	34.23	9.96
PUFAs	60.29	59.52	58.14	44.94	53.04

NDF—neutral detergent fiber; ADF—acid detergent fiber; ADL—acid detergent lignin; SFAs—saturated fatty acids; UFAs—unsaturated fatty acids; MUFAs—monounsaturated fatty acids; PUFAs—polyunsaturated fatty acids.

**Table 2:** Saturated fatty acid profile of intramuscular fat in the *Longissimus thoracis et lumborum* (LTL) muscle and leg muscles (LM) of New Zealand White rabbits.

Item	Muscles	Production System		SEM	*p*-Value
		Intensive (IPS)	Extensive (EPS)		
C12:0	LTL	1.54	1.75	0.137	0.468
	LM	1.24	2.08	0.313	0.192
C14:0	LTL	4.26	4.72	0.201	0.268
	LM	4.24	5.55	0.451	0.149
C15:0	LTL	0.95 ^b^	1.20 ^a^	0.057	0.018
	LM	1.05	1.17	0.062	0.352
C16:0	LTL	32.05	33.94	0.693	0.179
	LM	33.63	34.86	0.783	0.448
C17:0	LTL	0.94	0.97	0.072	0.821
	LM	0.99	1.00	0.046	0.879
C18:0	LTL	11.36	12.84	0.486	0.131
	LM	11.46	10.70	0.431	0.398
C20:0	LTL	0.51	0.49	0.041	0.813
	LM	0.31	0.30	0.017	0.624
SFAs	LTL	51.60 ^b^	55.92 ^a^	1.068	0.039
	LM	52.91	55.66	1.354	0.326

SEM—standard error of the mean; SFAs—saturated fatty acids. Values in rows followed by different superscript letters are significantly different: ^ab^—*p* ≤ 0.05.

**Table 3:** Unsaturated fatty acid profile of intramuscular fat in the *Longissimus thoracis et lumborum* (LTL) muscle and leg muscles (LM) of New Zealand White rabbits.

Item	Muscles	Production System		SEM	*p*-Value
		Intensive (IPS)	Extensive (EPS)		
C 14:1	LTL	0.15	0.22	0.032	0.281
	LM	0.16	0.22	0.026	0.252
C 16:1	LTL	2.94 ^b^	3.98 ^a^	0.190	0.003
	LM	2.86	3.15	0.148	0.332
C 17:1	LTL	0.24	0.28	0.051	0.709
	LM	0.27	0.28	0.027	0.867
C 18:1	LTL	21.69	20.71	0.282	0.083
	LM	20.29	19.36	0.432	0.297
C 18:2	LTL	18.31	15.19	0.873	0.072
	LM	17.86	16.44	1.004	0.496
C 18:3	LTL	2.84	2.17	0.277	0.242
	LM	3.64	3.22	0.546	0.716
C 20:1	LTL	0.32	0.31	0.044	0.947
	LM	0.38	0.27	0.043	0.210
C 20:4	LTL	1.90 ^a^	1.21 ^b^	0.114	<0.001
	LM	1.62	1.39	0.186	0.544
MUFAs	LTL	25.35	25.52	0.318	0.798
	LM	23.96	23.29	0.444	0.467
PUFAs	LTL	23.05 ^a^	18.575 ^b^	0.996	0.019
	LM	23.13	21.05	1.426	0.484
UFAs	LTL	48.40 ^a^	44.08 ^b^	1.068	0.039
	LM	47.09	44.34	1.354	0.326

SEM—standard error of the mean; MUFAs—monounsaturated fatty acids; PUFAs—polyunsaturated fatty acids; UFAs—unsaturated fatty acids (UFAs = MUFAs + PUFAs). Values in rows followed by different superscript letters are significantly different: ^ab^—*p* ≤ 0.05.

**Table 4:** Indicators of the nutritional value of intramuscular fat in the *Longissimus thoracis et lumborum* (LTL) muscle and leg muscles (LM) of New Zealand White rabbits.

Item	Muscles	Production System		SEM	*p*-Value
		Intensive (IPS)	Extensive (EPS)		
MUFA/SFA ratio	LTL	0.49	0.46	0.013	0.202
	LM	0.46	0.42	0.014	0.287
UFA/SFA ratio	LTL	0.94 ^a^	0.80 ^b^	0.036	0.045
	LM	0.90	0.82	0.044	0.352
PUFA/SFA ratio	LTL	0.45 ^a^	0.34 ^b^	0.025	0.026
	LM	0.45	0.39	0.033	0.441
DFAs	LTL	59.76 ^a^	56.92 ^b^	0.706	0.040
	LM	58.54	55.05	1.338	0.200
OFAs	LTL	40.24 ^b^	43.08 ^a^	0.706	0.040
	LM	41.46	44.95	1.338	0.200
DFA/OFA ratio	LTL	1.49 ^a^	1.33 ^b^	0.040	0.048
	LM	1.43	1.27	0.067	0.241
EFAs	LTL	21.15 ^a^	17.36 ^b^	0.929	0.037
	LM	21.50	19.67	1.268	0.486
Nutritional value *	LTL	1.04	1.00	0.021	0.361
	LM	0.95	0.87	0.029	0.209

SEM—standard error of the mean; UFAs—unsaturated fatty acids (MUFAs + PUFAs); SFAs—saturated fatty acids; MUFAs—monounsaturated fatty acids; PUFAs—polyunsaturated fatty acids; DFAs—hypocholesterolemic fatty acids (UFAs + C18:0); OFAs—hypercholesterolemic fatty acids (SFAs—C18:0); EFAs—essential fatty acids (C18:2 + C18:3); * (C18:0 + C18:1)/C16:0). Values in rows followed by different superscript letters are significantly different: ^ab^—*p* ≤ 0.05.

## Data Availability

Data available on reasonable request.
